# Identification of the genetic basis of the duck growth rate in multiple growth stages using genome-wide association analysis

**DOI:** 10.1186/s12864-023-09302-8

**Published:** 2023-05-26

**Authors:** Yang Xi, Qifan Wu, Yutian Zeng, Jingjing Qi, Junpeng Li, Hua He, Hengyong Xu, Jiwei Hu, Xiping Yan, Lili Bai, Chunchun Han, Shenqiang Hu, Jiwen Wang, Hehe Liu, Liang Li

**Affiliations:** Farm Animal Genetic Resources Exploration and Innovation Key Laboratory of Sichuan Province, College of Animal Science and Technology, Sichuan Agricultural University, Chengdu, People’s Republic of China

**Keywords:** Duck, GWAS, Growth rate

## Abstract

**Background:**

The genetic locus responsible for duck body size has been fully explained before, but the growth trait-related genetic basis is still waiting to be explored. For example, the genetic site related to growth rate, an important economic trait affecting marketing weight and feeding cost, is still unclear. Here, we performed genome wide association study (GWAS) to identify growth rate-associated genes and mutations.

**Result:**

In the current study, the body weight data of 358 ducks were recorded every 10 days from hatching to 120 days of age. According to the growth curve, we evaluated the relative and absolute growth rates (RGR and AGR) of 5 stages during the early rapid growth period. GWAS results for RGRs identified 31 significant SNPs on autosomes, and these SNPs were annotated by 24 protein-coding genes. Fourteen autosomal SNPs were significantly associated with AGRs. In addition, 4 shared significant SNPs were identified as having an association with both AGR and RGR, which were Chr2: 11483045 C>T, Chr2: 13750217 G>A, Chr2: 42508231 G>A and Chr2: 43644612 C>T. Among them, Chr2: 11483045 C>T, Chr2: 42508231 G>A, and Chr2: 43644612 C>T were annotated by *ASAP1*, *LYN* and *CABYR,* respectively. *ASAP1* and *LYN* have already been proven to play roles in the growth and development of other species. In addition, we genotyped every duck using the most significant SNP (Chr2: 42508231 G>A) and compared the growth rate difference among each genotype population. The results showed that the growth rates of individuals carrying the Chr2: 42508231 A allele were significantly lower than those without this allele. Moreover, the results of the Mendelian randomization (MR) analysis supported the idea that the growth rate and birth weight had a causal effect on the adult body weight, with the growth rate having a greater effect size.

**Conclusion:**

In this study, 41 SNPs significantly related to growth rate were identified. In addition, we considered that the *ASAP1* and *LYN* genes are essential candidate genes affecting the duck growth rate. The growth rate also showed the potential to be used as a reliable predictor of adult weight, providing a theoretical reference for preselection.

**Supplementary Information:**

The online version contains supplementary material available at 10.1186/s12864-023-09302-8.

## Background

Meat is an important component of a healthy and well-balanced diet, providing high-quality protein and fat sources for the body [1]. However, with the rapid growth of the global population, determining ways to ensure an adequate food supply has become the focus of scientists [2]. Improving the growth performance of animals is perhaps one of the most direct methods. Growth performance can be affected by various factors, such as feed nutrition [3], feeding mode [4], and environmental factors [5]. In addition to those mentioned above, genetic factors are also one of the factors that cannot be ignored. The growth performance in different breeds of the same species often presents obvious differences [6, 7]. Therefore, improving animal growth performance at the genetic level has always been a major goal for breeders.

Originally, the candidate gene approach was a reliable way of identifying molecular markers and conducting genetic improvement [8–10]. However, with the present understanding of genetic research, the candidate gene approach has been shown to have some limitations in explaining complex traits. Fortunately, the maturity of high-throughput sequencing technology and the wide application of the genome-wide association study (GWAS) make it possible to comprehensively identify genetic loci affecting complex traits such as growth rate. To date, in farm animals such as pigs, cattle, and chickens, over 1,000 quantitative trait loci (QTLs) from the animal QTL database have been identified to be associated with growth-related traits such as body weight gain and average daily gain. A growth rate-related GWAS in a pig population including four breeds identified nine growth-related candidate genes [11]. A recent GWAS on growth traits in the Duroc population found that the most significant genetic region could explain 18.95% of the variation in average daily gain [12]. The above studies advanced our understanding of pig growth-related traits. In the Brahman cattle population, 167 and 262 significant SNPs were identified to be significantly associated with mature weight and maturity rate, respectively, and several candidate genes related to muscle and bone development were found [13]. In poultry, research on genetic mutations and candidate genes related to growth traits has also been ongoing. Recently, a study identified the *TGF3* gene as a genetic marker for chicken growth traits by candidate gene approach [14]. However, a survey of the chicken pangenome has made great progress. Wang et al. successfully identified a deletion mutation in the promoter region of the *IGF2BP1* gene as a causal mutation affecting growth traits such as chicken body size through GWAS and fine mapping methods [15]. Molecular marker-assisted selection using this mutation will greatly help to produce high-productivity chicken breeds. Interestingly, a few years ago, the same gene (*IGF2BP1*) in ducks was also identified as the gene responsible for affecting duck body size [16]. Subsequent studies also conducted a GWAS on Peking ducks and identified QTLs related to growth and feeding efficiency [2].

The duck is an important economic animal, especially in Asian countries. Although *IGF2BP1* is responsible for body size in ducks, there are still many unknown growth-related genetic loci to be identified, such as the genetic site related to growth rate, which is one of the essential indices to evaluate growth performance and an important economic trait affecting the marketing age [17]. Therefore, in this study, we studied 358 ducks (*Anas domesticus*) and collected body weight data every 10 days from egg hatching (Day 1) to 120 days of age. A GWAS was conducted to identify genetic loci related to growth performance. The results deepen our understanding of the genetic basis of duck growth.

## Results

### Growth performance evaluation

**Fig. 1:**
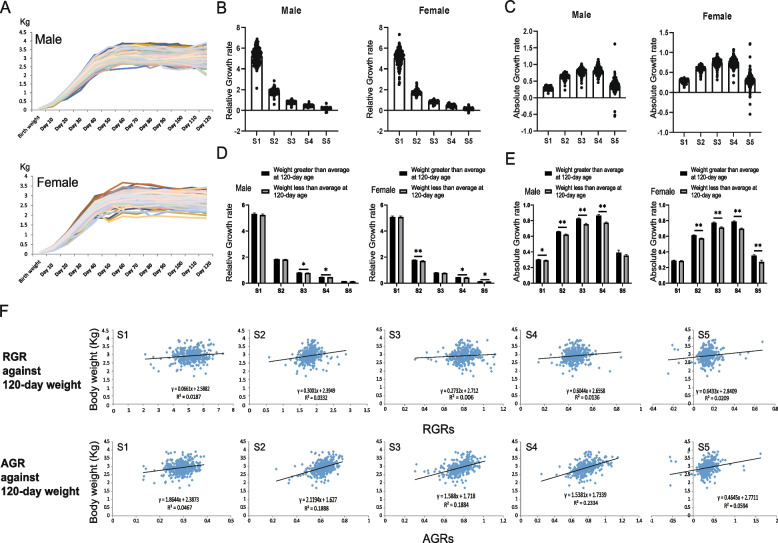
Growth rate evaluation. **A** Male and female ducks' body weight growth curve from birth to 120-day old. The X-axis and Y-axis showed the ages and body weights, respectively. **B **and **C** The RGRs **B** AGRs **C** of S1-S5 growth stages. The dots in the bar charts showed the RGR or AGR of one individual. The X-axis and Y-axis represent different growth stages and growth rates data. **D **and **E** Comparison of RGRs and AGRs between the ducks heavier and lower than average body weight in 120-day old. The significance was shown as * (*P* < 0.05) and ** (*P* < 0.01). The contents represented by the horizontal and vertical coordinates were consistent with Fig. **B** and **C**. **F** The correlation analysis between growth rates and body weight data at 120-day old. The fitting curves and the Pearson correlation coefficient (R^2^) could reflect the correlation degree. The abscissa of each scatter plot represents the growth rates, and the ordinate represents 120-day body weight

To better evaluate the growth rate of the research population, we collected the body weight data of each duck every 10 days from hatching to 120 days of age and then constructed the growth curves of the male and female populations (Fig. 1A). As shown in the results, both male and female ducks were in a rapid growth stage before 40 days, but the growth rate gradually slowed down after 40 days, and the weight was stable after 50 days. Therefore, we calculated the RGRs and AGRs of 5 stages of all ducks before the age of 50 days, which were S1 (from birth weight to 10-day weight), S2 (from 10-day weight to 20-day weight), S3 (from 20-day weight to 30-day weight), S4 (from 30-day weight to 40-day weight) and S5 (from 40-day weight to 50-day weight). The RGR and AGR patterns showed a slight difference in the growth peak. The RGRs of the population reached a maximum at the beginning and then decreased gradually, while the peak of AGR appeared at S4. Both fell to the lowest level and showed a negative growth for the first time at S5 (Fig. 1B and C). Then, the relationship between growth rate and final adult body weight was investigated. With the body weight at 120 days as a reference, we compared the RGR and AGR differences between individuals with a heavier weight than average and a lower than average weight. At this stage, the weight of male and female ducks had already peaked and did not increase, indicating that the body weights of the ducks had attained somatic maturity. The results showed that the RGR and AGR were higher among heavier ducks at the age of 120 days (Fig. 1D and E). In particular, the AGR showed extremely significant differences (*P* < 0.01) at almost every growth stage (Fig. 1E). The correlation analysis also reflected similar results. AGRs and RGRs at each stage all showed varying degrees of positive correlation with the 50-day weight, while the RGRs could only explain the variation in 120-day body weight by 0.06% - 3.32%. For AGRs, this range was increased to 4.67% - 23.34% (Fig. 1F). The above results indicated that the AGR had a higher correlation with the final stable weight after the rapid growth stage of ducks and thus has the potential to be an important predictor for the early prediction of market weight.

### GWAS based on RGR

A total of 4,237,004 filtered SNPs were obtained and used for the subsequent GWAS with a minor allele frequency (MAF) > 0.03 and a maximum missing rate < 0.1 among all 358 individuals from 5 synthetic lines (SL1-SL5) (Figure S1). Then, principal component analysis (PCA) was conducted to analyse the population structure. No obvious population stratification was found among the different synthetic lines, while 3 subpopulations were identified in SL1 ducks (Figure S2). Therefore, we used the first 3 principal components as the covariates to correct the influence of genetic backgrounds in the GWAS.

**Fig. 2:**
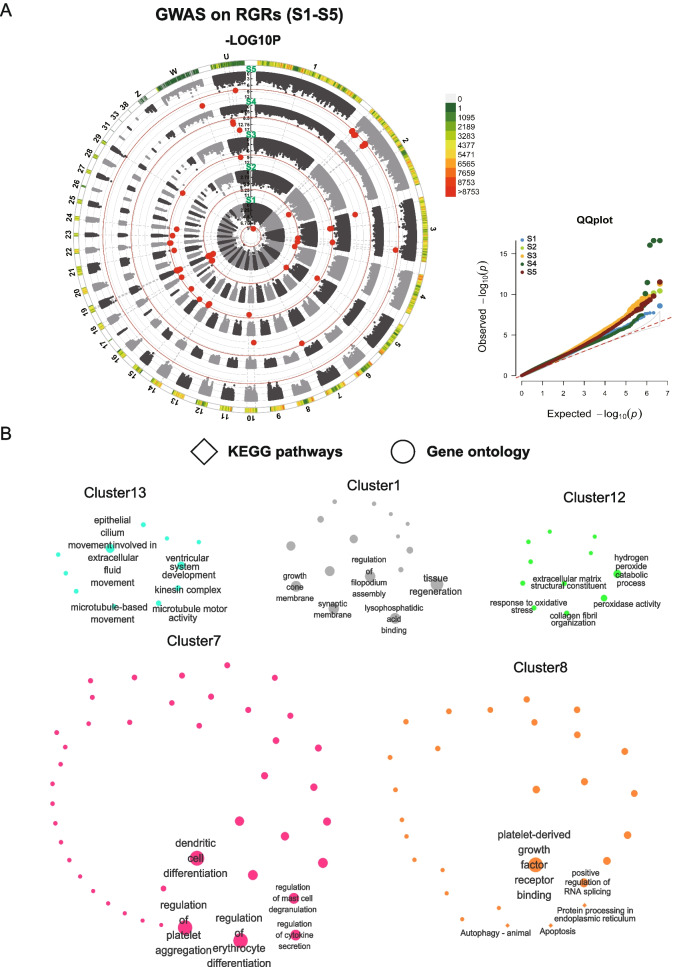
SNPs associated with the RGRs. **A** GWAS between genotypes and RGRs (left) and QQ plots (right). The red lines represent the Bonferroni corrected significance threshold (-log10*P* = 8.59). And the red dots were the significant SNPs that crossed the threshold. GWAS results of RGRs from S1 – S5 were shown from inside to outside. **B** The KEGG pathways (diamonds) and GO functions (circles) in which significant related SNPs-related genes are enriched. Each node represents an enriched term, and the node colour represents different clusters. The higher the enriched factors, the smaller the node sizes are. Only the first 5 terms with highest enrich ratios in each cluster were marked with text

First, a GWAS based on the RGR was conducted. There were 31 SNPs identified on autosomes with a significant association with the RGRs of different growth stages by the criteria of –log_10_*P* > 8.59 (0.05/total SNP number) (Fig.  2A**, **Table S1). We carried out annotation analysis on these SNPs to investigate the genes related to these mutations (Table S1). After removing the pseudogenes and unannotated ncRNAs, a total of 24 protein-coding genes were identified, including a gene widely related to growth, *growth-associated protein-43* (*GAP43*), which was related to the SNP significantly associated with the RGR at the S1 stage (Table S1), implying that this gene might participate in the early growth of ducklings. KEGG enrichment analysis (Table S2) was conducted for the other identified genes to explore the potential candidate genes that determine the duck growth rate. Only 6 known KEGG pathways in which the *ERN1*, *GFPT2,* and *ASAP1* genes participate were identified (Table S2). In addition, the GO analysis provided more information. We clustered the KEGG and GO terms with similar functions (Table S2), and the top 5 clusters contained most pathways and GO functions (Fig. 2B). Multiple potential growth-related pathways and clusters were identified, including the regulation of growth and nervous system development in Cluster 1, in which the *GAP43* gene was enriched (Fig. 2B**,** Table S2). In addition, we also found genes regulating microtubule development (Cluster 2, *CAMSAP1*), bone mineralization (Cluster 3, *ANKH*), cell proliferation and differentiation (Cluster 7: *LYN*), and adipose tissue development (Cluster 15, *EBF2*) (Table S2). Above all, these results indicated that the genes related to the significant SNP might play essential roles in duck growth and weight determination.

### GWAS based on AGR

**Fig. 3:**
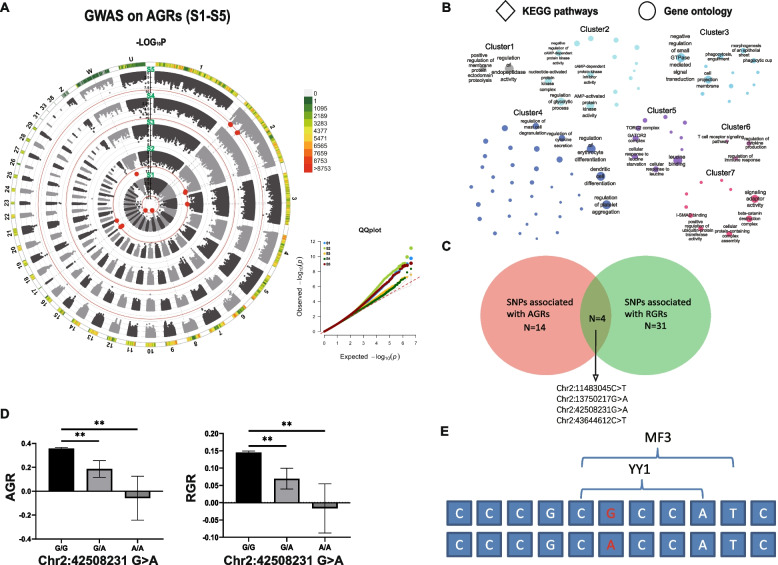
SNPs associated with the AGRs. **A** GWAS between genotypes and AGRs (left) and QQ plots (right). The red lines represent the Bonferroni corrected significance threshold (-log10*P* = 8.59). And the red dots were the significant SNPs that crossed the threshold. GWAS results of AGRs from S1 – S5 were shown from inside to outside. **B** The KEGG pathways (diamonds) and GO functions (circles) in which significant related SNPs-related genes are enriched. Each node represents an enriched term, and the node colour represents different clusters. The higher the enriched factors, the smaller the node sizes are. Same as Fig. 2, only the first 5 terms with highest enrich ratios in each cluster were marked with text. **C** The shared significant SNPs between the AGRs and RGRs GWAS results. **D** Phenotypic comparisons between different genotypes based on the most significant SNP, Chr2: 42508231 G>A. The X-axis represents the different genotypes, and the Y-axis shows the AGR and RGR in the S5 growth stage. **E** Changes in the binding sites of Chr2: 42508231 G>A were demonstrated. The sequence used in the analysis was 5 bp of the left and right flanks of the mutation site

Then, we continued to analyse the genetic loci associated with AGR. In the GWAS results of S1-S5, only three stages of AGR (S1, S2, and S5) were found to have significantly associated SNPs (Fig. 3A). Fourteen autosomal SNPs were found to be significantly associated with AGRs at the above mentioned stages (Table S3). We carried out gene annotation for these SNPs, and the related genes were enriched in various signalling pathways and gene functions by KEGG and GO analysis (Fig. 3B, Table S3, Table S4). It is worth noting that the pathways and functions enriched in Cluster 4 were highly coincident with Cluster 7 of the previous RGR-related gene enrichment result, which is mainly associated with cell proliferation and differentiation (Table S2 and Table S4). Therefore, we investigated the intersection of AGR- and RGR-associated SNPs. A total of 4 shared SNPs were found to be significantly associated with the AGR and RGR in the S5 growth stage and were all located on chromosome 2 (Fig. 3C). The linkage disequilibrium (LD) analysis identified two potential linkage intervals, Block1 (Chr2: 11483045 C>T & Chr2: 13750217 G>A) and Block2 (Chr2: 42508231 G>A & Chr2: 43644612 C>T), among these 4 SNPs (Figure S3). The annotated genes of these mutations were *ASAP1* (Chr2: 11483045 C>T), *LYN* (Chr2: 42508231 G>A), and *CABYR* (Chr2: 43644612 C>T) (Table S1 and Table S3). Among these, Chr2: 42508231 G>A had the highest –log_10_*P* value in the GWAS based on both AGR and RGR (Table S1 and Table S3) and was located in the intron region of the *LYN* gene, which was reported to participate in epithelial and haematopoietic cell functions, including cell differentiation, growth, proliferation, and migration (Table S2 and Table S4). We then genotyped the Chr2: 42508231 G>A mutation and investigated the growth rates of individuals with different genotypes. The results showed that AGR and RGR at the S5 stage were significantly lower in the ducks carrying the Chr2: 42508231 A allele (Fig. 3D). According to the TF binding site prediction of the mutation, two transcription regulator binding sites (YY1, MF3) were found to be possibly lost after the mutation of Chr2: 42508231 (Fig. 3E).

### Growth rate is one of the causal factors determining adult body weight

**Fig. 4:**
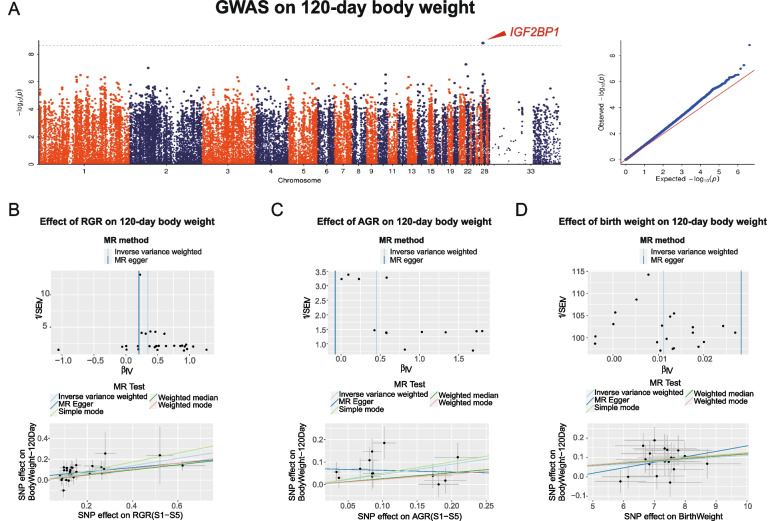
Causal effect of growth rate and birth weight on the adult body weight. **A** GWAS based on the 120-day body weight. The gray line represents the Bonferroni corrected significance threshold (-log10*P* = 8.59). The x-axis shows the physical positions of each marker along the chromosome, and the y-axis shows the - log10*P* values of SNPs. **B**, **C **and **D** Funnel plot displaying instrument strength (y-axis) plotted against causal effect estimates (x-axis) for SNPs associated with RGR **B**, AGR **C**, and birth weight **D**. Scatter plots displaying estimates of the association between each SNP and 120-day body weight (y-axis) against estimates of the association between each SNP and RGR **B**, AGR **C**, and birth weight **D** (x-axis). The error bars on each of the points indicated the 95% CI. The causal effect estimates of Inverse variance weighted, MR Egger, Simple mode, Weighted median, and Weighted mode were represented by solid lines in different colours

In the first part of the results, we found correlations between AGRs and body weight at 120 days of age. Therefore, we intended to explore the causal relationship between growth rate and adult weight and whether individuals with a high growth rate will grow heavier. We conducted a GWAS on the body weight phenotypic data at each stage. From 70 days of age, a stable signal appeared on chromosome 28. By gene annotations, this signal corresponded to the gene *IGF2BP1*, which was proven to be associated with duck body size in a previous study [16]. However, it was interesting that this signal was not found in any GWAS results of body weight or growth rates at the earlier development stages until the age of 70 days (Figure S4 - S15, Fig. 4A). It seemed that there was no obvious relationship between the adult weight and the growth rate of ducks from the simple GWAS results. To examine this further, we took all SNP sites related to growth rate as instrumental variables after removing the SNPs with mutual LD relationships and conducted Mendelian randomization analysis. First, we analysed the effect of RGRs on 120-day body weight. A total of 29 filtered SNPs were involved in the subsequent analysis. Little indication for the existence of directional horizontal pleiotropy for the RGRs was observed by visual inspection of the funnel plot, which was consistent with the results of the directional horizontal pleiotropy test (*P* > 0.05) (Fig. 4B**,** Table S5). The TwoSampleMR results showed that as RGR increased, the 120-day body weight increased as well by using MR-egger (Beta = 0.21, *P* = 3.04E-02; OR = 1.23, 95% CI: [1.03, 1.47]), weighted median (Beta = 0.24, *P* = 3.29E-03; OR = 1.28, 95% CI: [1.08, 1.50]), IVW (Beta = 0.35, *P* = 1.71E-10; OR = 1.41, 95% CI: [1.27, 1.57]), simple mode (Beta = 0.43, *P* = 8.36E-03; OR = 1.54, 95% CI: [1.14, 2.07]), and weighted mode (Beta = 0.27, *P* = 1.46E-03; OR = 1.31, 95% CI: [1.13, 1.52]). In addition, the above five MR methods all showed that the significant promoting effect of a single SNP on the 120-day body weight increased with the single SNP effect on the RGRs (Fig. 4B**,** Table S6). The same analysis process was performed on AGRs as well. A potential horizontal pleiotropy was found. However, the IVW method (Beta = 0.45, *P* = 1.80E-03; OR = 1.57, 95% CI: [1.18, 2.08]) still showed a significant positive causal effect of AGRs on the 120-day body weight (Fig. 4C**, **Table S5, Table S6). The above results indicated that the growth rate was indeed one of the essential causes affecting adult weight. Then, we further explored the causal effect of birth weight on the 120-day body weight. The results also supported the idea that birth weight had a significant causal impact on 120-day body weight by all 5 analysis methods (Fig. 4D**, **Table S6). However, the effect value (Beta: about 0.01) of birth weight on adult weight was much lower than that of the growth rate, implying that the growth rate was more able to predict the adult body than the birth weight.

## Discussion

Body growth is a physiological process affected by multiple complex factors. In the present study, we identified multiple SNPs associated with duck growth weight in 358 ducks. Among all the SNPs identified, 4 were shared in both the AGR and RGR GWAS results, and these SNPs were annotated by 3 genes: *ASAP1* (Chr2: 11483045 C>T), *CABYR* (Chr2: 43644612 C>T) and *LYN* (Chr2: 42508231 G>A). ASAP1 is a GTPase-activating protein (GAP) that can negatively regulate ADP ribosylation factor (Arf). The Arf family is mainly involved in regulating membrane vesicle transport and the cytoskeleton [18]. Moreover, Arf was proven to be associated with early embryonic development in mice and regulates the growth, migration, and invasion of cancer cells [19, 20]. Studies on the *ASAP1* gene have shown that this gene also plays an essential role in regulating growth and development. In mice, loss of the ASAP1 gene can lead to growth retardation, delayed ossification, adipocyte development, and fat depot formation reduction [21]. The SNPs related to the *ASAP1* gene have also been associated with production and growth traits in cattle [22, 23]. The *CABYR* gene has been widely proven to be related to sperm morphology and sperm development [24, 25], but no research has yet shown that this gene is involved in the growth performance of animals or plants. However, the other gene located in the same LD block as *CABYR*, *LYN*, which belongs to the Src family of intracellular membrane-associated tyrosine kinases, is an important candidate gene for growth traits in many farm animals. Like most genes regulating growth traits, LYN plays an essential role in the occurrence and development of cancers [26–28] since it can function in the signal transduction of growth factor receptors [29]. LYN is a vital candidate gene affecting human height [30]. In animals, the LYN gene was identified to be associated with various growth traits of cattle, such as feed intake, growth, body weight, and birth weight. [31–34]. In chickens, the *LYN* gene was identified as being associated with comb development [35]. One study explained that the possible route by which the *LYN* gene affects growth traits is by regulating the intestinal microbial composition, 2 SNPs were identified in *LYN* and *PLAG1* that were significantly associated with *Methanobacterium,* which had a higher abundance in high-weight female chickens [36]. In addition, the *YY1* gene, which was predicted to lose a binding site at the regulatory region of the *LYN* gene, was previously proven to participate in fat metabolism. The loss of the YY1 protein in mice can protect against diet-induced obesity and increase energy consumption [37]. From the prediction result and similarity of biological function, a hypothesis of the potential regulatory relationship between the YY1 transcription factor and the *LYN* gene could be debated. However, the actual situation still needs further verification.

A previous study determined that *IGF2BP1* is the major gene affecting the body shape of ducks [16], while in the present study, *IGF2BP1* signals were only identified in the GWAS results of body weight after 60 days of age. This indicates that the *IGF2BP1*-related mutation may not be able to evaluate early growth traits very well. However, early growth trait indicators, such as birth weight, are usually used to predict adult body weight and conduct early selection. Meanwhile, it is unclear whether ducks' birth weight affects their subsequent growth performance. Studies in other species are still controversial as well. Some researchers have suggested that the birth weight of pigs has a greater impact on pig growth performance after weaning [38], while another study suggested that birth weight did not always determine subsequent growth performance [39]. The MR analysis showed that both birth weight and growth rate had causal effects on the 120-day slaughter weight, while the impact of birth weight on slaughter weight was slight. From the aspect of effect size, it could be even said that the duck birth weight is not so important to postnatal growth performance which is similar to the research result in other terrestrial vertebrates [40]. In contrast, the early growth rate can be used as a more reliable predictor of final adult body weight. In addition, these results indirectly indicated that SNPs that affect growth rate also play a role in duck adult body weight as minor effect sites to some extent.

Based on the above description, we considered the *ASAP1* and *LYN* genes to be important candidate genes affecting duck growth rates. Moreover, the SNPs we identified could be used as potential molecular markers to select the growth rate phenotype, leading to improvements in the marketing age and growth performance.

## Materials and methods

### Animal samples and phenotype collection

A total of 358 ducks (*Anas domesticus*) (Male: *n* = 168; Female: *n* = 180; Record missing: *n* = 10) from 5 synthetic lines (SL1-SL5) bred by poultry breeding farm of Sichuan Agricultural University were included in the main research materials in the present study. The duck genetic background of each population is relatively independent, and there is no cross between them. The DNA samples were extracted from the blood cells of all 358 individuals by phenol-chloroform extraction method for genomic re-sequencing. The experimental ducks were reared in the brooding room for 0-14 days. The feeding density was 30 ducks / m^2^, and they were free-fed all day. During this time, the lighting time was 24 hours a day. At the age of 15~120 days, the ducks were fed in the ground flat breeding house, and the feeding density and lighting time was 1.5 ducks / m^2^ and 8~10 hours/day. At the age of 15-60 days, all ducks had 12 hours of free feeding. After 60 days of age, the feeding restriction was conducted, and the average daily intake was 0.13 Kg/duck. During the feeding period, each duck shall be weighed every ten days. And the gain of body weight between two weighing times is defined as AGR. The ratio of AGR to the previous weight is considered as the RGR. The calculation formulas are as follows (W_0_: measured weight at last time; W_1_: measured weight at next time):

$$\mathrm{AGR}={\mathrm{W}}_{1}- {\mathrm{W}}_{0}$$

$$\mathrm{RGR}=\frac{{\mathrm{W}}_{1}- {\mathrm{W}}_{0}}{{\mathrm{W}}_{0}}\times 100\%$$

All ducks were euthanized at 120 days old by inhaling carbon dioxide and cervical dislocation, which performed by competent personnel who experienced and correctly applied the technique. The Animal Welfare Committee approved the protocols for all Sichuan Agricultural University animal experiments, and all methods strictly obeyed the Guide for the Care and Use of Agricultural Animals in Research and Teaching . The metadata of all samples were collected in the Table S7.

### Genomic SNP identification

The DNA samples were all extracted from duck whole blood using a standard Phenol-Chloroform extraction protocol. The quality and quantity of DNA were examined using a NanoDrop2000 device and by agarose gel electrophoresis. After the examinations, standard procedures generated paired-end libraries for each eligible sample. The average insert size was 500 bp, and the average read length was 150 bp. All libraries were sequenced on an Illumina® HiSeq X Ten or HiSeq 4000 platform to an average raw read sequence coverage of ×5 for the duck population. The depth ensured the accuracy of variant calling and genotyping and met the requirements for population genetic analyses. Raw sequence data were mapped to the duck reference genome (ZJU 1.0, RefSeq assembly accession: GCF_015476345.1) with Burrows-Wheeler alignment (BWA aln) using the default parameters [41]. SNP calling was performed exclusively using GATK (version 3.5) [42], and the output was further filtered using VCFtools (version 0.1.15) [43]. The SNPs were filtered based on the following criteria: (1) the SNPs had to have a minor allele frequency > 0.03; (2) the maximum missing rate was < 0.1; and (3) the SNPs had only two alleles; (4) SNPs with heterozygosity greater than 80% will be removed. For all 358 individuals, over 4 million SNPs were finally obtained and used for the subsequent analysis.

### PCA analysis

PCA was performed based on all SNPs using GCTA tool [44]. This software applies PCA to genetic data to analyse the population structure. The figures were plotted using the first, second and third principal components with R package ggplot2 (version: 3.3.6).

### GWAS

Population structure and cryptic relatedness were used to minimize false positives and to increase statistical power. The mixed linear model program Emmax was used for the association analysis [45]. The first three principal component values (PCA eigenvectors) derived from the whole-genome SNPs and the forward/ backward crosses were fitted as fixed effects in the mixed model to correct population stratification [46]. The random effect was the kinship matrix estimated from all individual whole-genome SNPs. We defined the whole-genome significance cutoff as the Bonferroni threshold, which is 0.05/total SNPs (−log_10_*P* =8.59).

### Two-sample mendelian randomization analysis

Two-sample MR analyses were performed using the GWAS results data. After removing the LD relationship, all SNPs associated with AGRs or RGRs and the top 20 SNPs associated with birth weight were chosen as the instruments for the MR analyses. To obtain estimates of the causal effect of adult weight, we performed Inverse variance weighted, MR Egger, Simple mode, Weighted median, and Weighted mode analysis to evaluate the causal effect of growth rate and birth weight on the adult body weight. Analyses were performed using the TwoSampleMR R package of MR-Base [47].

### Data statistics and other bioinformatics tools

All correlation analysis was performed using the Pearson correlation method. The unpaired t-test was used to analyse whether there were significant differences (*P* < 0.05). The KEGG and GO enrichment was conducted using KOBAS 3.0 online tools (http://bioinfo.org/kobas/genelist/). The trans-factor binding site prediction was performed by PROMO (http://alggen.lsi.upc.es/cgi-bin/promo_v3/promo/promoinit.cgi?dirDB=TF_8.3).

## Supplementary Information

**Additional file 1: Supplementary Table S1.** The significant SNPs associated with RGRs and the gene annotation.** Supplementary Table S2. **The KEGG and GO enrichment of RGR-associated genes.** Supplementary Table S3.** The significant SNPs associated with AGRs and the gene annotation. **Supplementary Table S4. **The KEGG and GO enrichment of AGR-associated genes.** Supplementary Table S5.** Heterogeneity and directional horizontal pleiotropy test of SNPs associated with AGRs, RGRs, and birth weight. **Supplementary Table S6.** The MR analysis results between exposures of AGR, RGR, birth weight and the outcome of the 120-day body weight. **Supplementary Table S7. **The metadata of all samples used in the research.

**Additional file 2: Supplementary Figure S1.** SNP distribution. The number of SNPs within a 1 Mb window size distribute across all chromosomes. The color from green to red represents the gradual increasing SNP number in each window. **Supplementary Figure S2.** Principal Component Analysis. PCA was conducted on the SNP information of each sample using the GCTA tool. The blue, red, green, cyan and black dots represent SL1 - SL5 populations, respectively. **Supplementary Figure S3.** Linkage disequilibrium analysis. The numbers in each diamond-shaped box represent the correlation coefficients R2 between two SNPs, and the R2 > 0.2 was considered to indicate linkage. **Supplementary Figure S4-S15.** GWAS based on body weight from birth (Figure S4) to 110 days old ( Figure S15).

## Data Availability

All Genomic re-sequencing raw data files were submitted to the SRA database (Accession: PRJNA907492 & PRJNA907501).

## Abbreviations

GWAS: Genome-wide association studies
QTLs: Quantitative trait loci
LD: Linkage disequilibrium
SNP: Single nucleotide polymorphism
MAF: Minor allele frequency
PCA: Principal component analysis
MR: Mendelian Randomization
KEGG: Kyoto Encyclopedia of Genes and Genomes
GO: Gene ontology
AGR: Absolute growth rate
RGR: Relative growth rate
CABYR: Calcium Binding Tyrosine Phosphorylation Regulated
ASAP1: Development and Differentiation-Enhancing Factor 1
LYN: LYN Proto-Oncogene, Src Family Tyrosine Kinase
IGF2BP1: Insulin-Like Growth Factor 2 MRNA Binding Protein 1
