# Supplementary material for: Identification of the genetic basis of the duck growth rate in multiple growth stages using genome-wide association analysis
Source: BMC Genomics. 2023 May 26;24:285. doi: 10.1186/s12864-023-09302-8 (PMC10223888; doi:10.1186/s12864-023-09302-8)
Supplement: Supplementary file 2 — Additional file 2: Supplementary Figure S1. SNP distribution. The number of SNPs within a 1 Mb window size distribute across all chromosomes. The color from green to red represents the gradual increasing SNP number in each window. Supplementary Figure S2. Principal Component Analysis. PCA was conducted on the SNP information of each sample using the GCTA tool. The blue, red, green, cyan and black dots represent SL1 - SL5 populations, respectively. Supplementary Figure S3. Linkage disequilibrium analysis. The numbers in each diamond-shaped box represent the correlation coefficients R2 between two SNPs, and the R2 > 0.2 was considered to indicate linkage. Supplementary Figure S4-S15. GWAS based on body weight from birth (Figure S4) to 110 days old ( Figure S15). [file 12864_2023_9302_MOESM2_ESM.docx]

**Figure S1 SNP distribution.** The number of SNPs within a 1 Mb window size distribute across all chromosomes. The color from green to red represents the gradual increasing SNP number in each window.

**Figure S2 Principal Component Analysis.** PCA was conducted on the SNP information of each sample using the GCTA tool. The blue, red, green, cyan and black dots represent SL1 - SL5 populations, respectively.


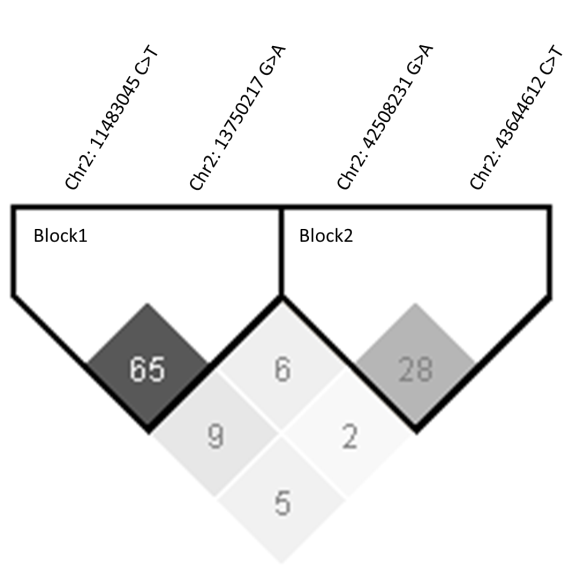


**Figure S3 The linkage disequilibrium analysis.** The numbers in each diamond-shape boxed represent the correlation coefficients R^2^ between two SNPs, and the R^2^ > 0.2 was considered to indicate linkage.


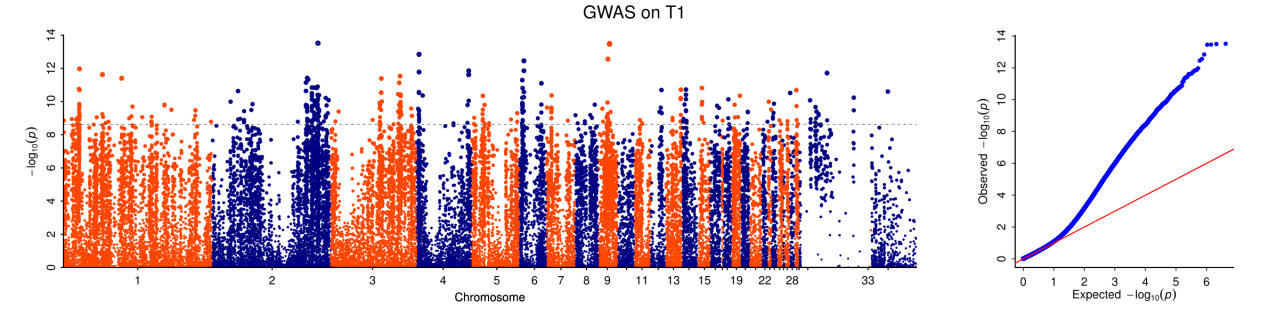


Figure S4 GWAS based on birth weight.


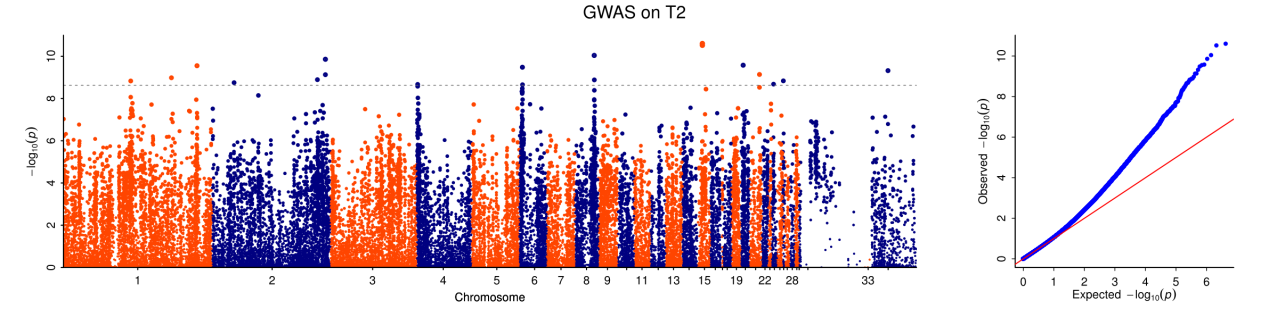


Figure S5 GWAS based on 10 days old.


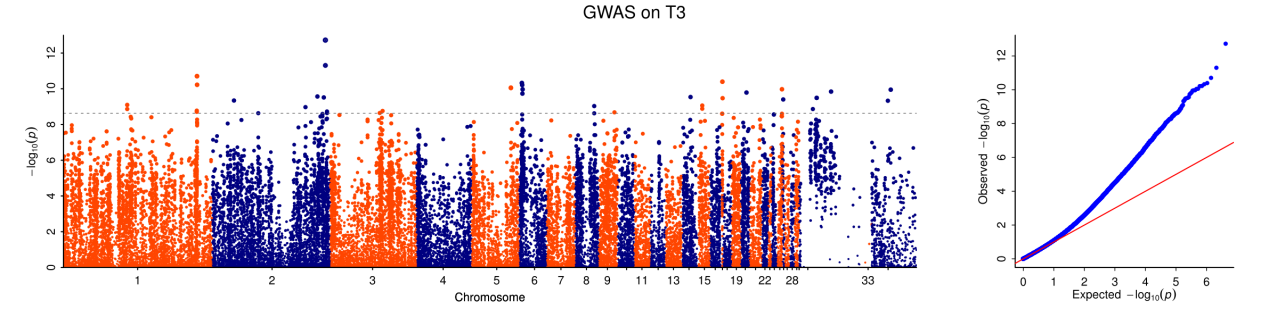


Figure S6 GWAS based on 20 days old.


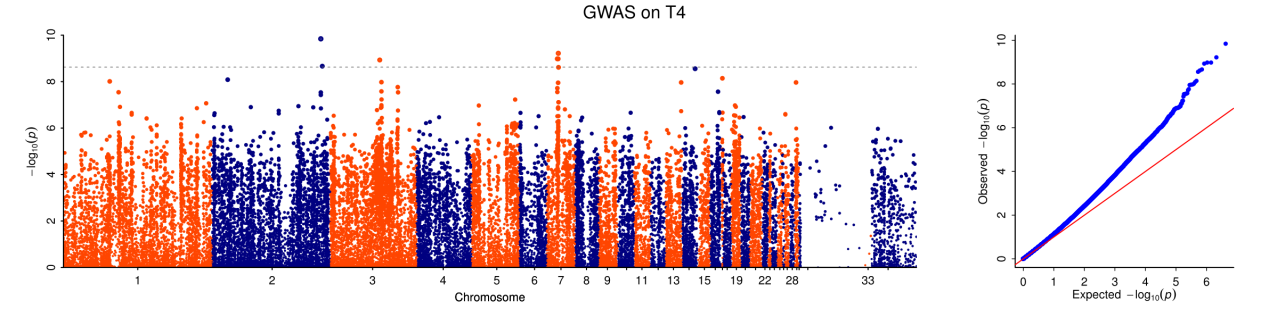


Figure S7 GWAS based on 30 days old.


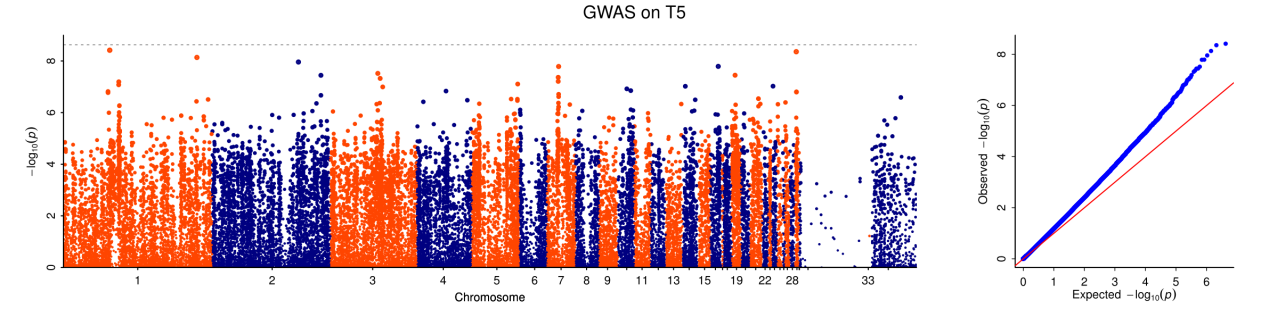


Figure S8 GWAS based on 40 days old.


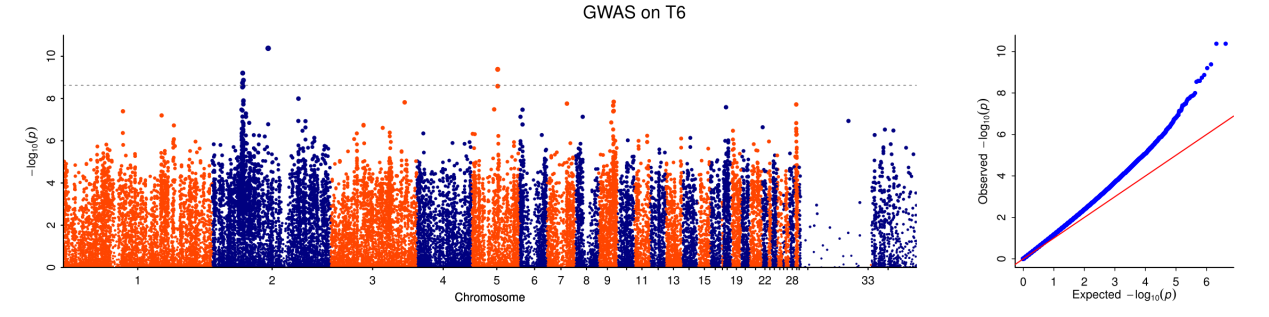


Figure S9 GWAS based on 50 days old.


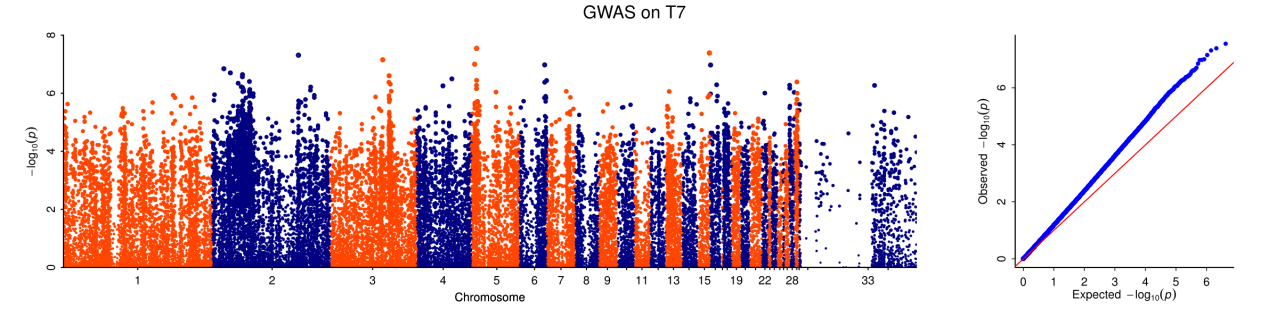


Figure S10 GWAS based on 60 days old.


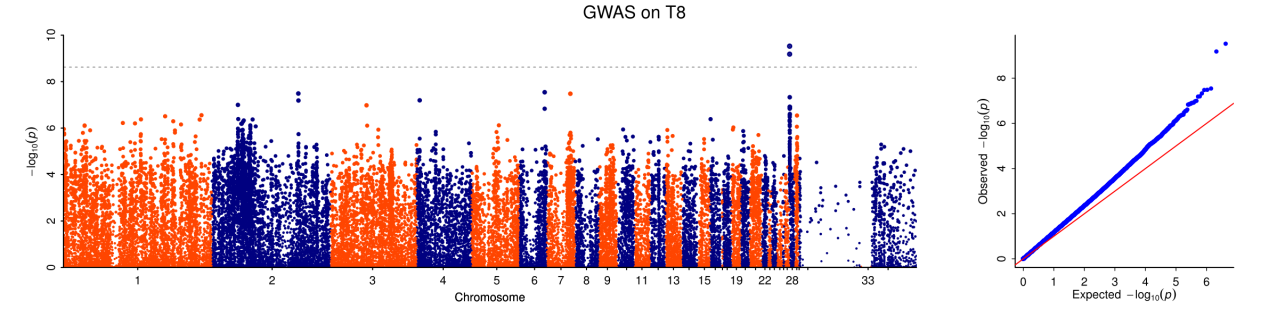


Figure S11 GWAS based on 70 days old.


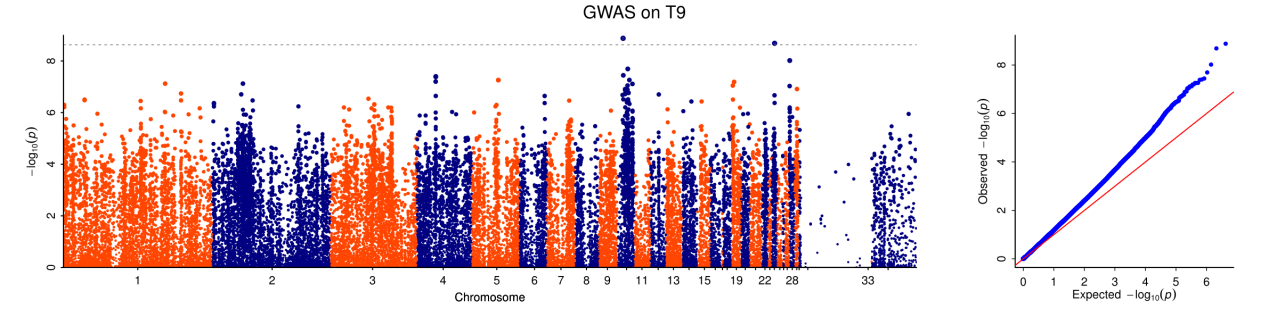


Figure S12 GWAS based on 80 days old.


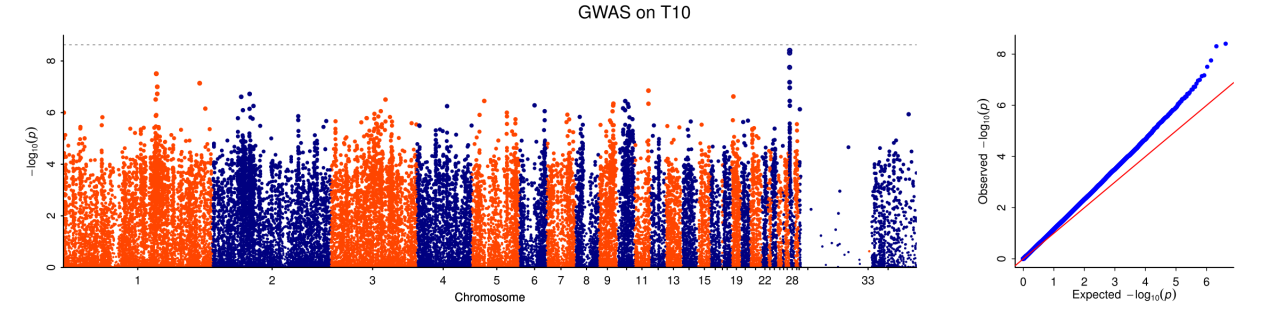


Figure S13 GWAS based on 90 days old.


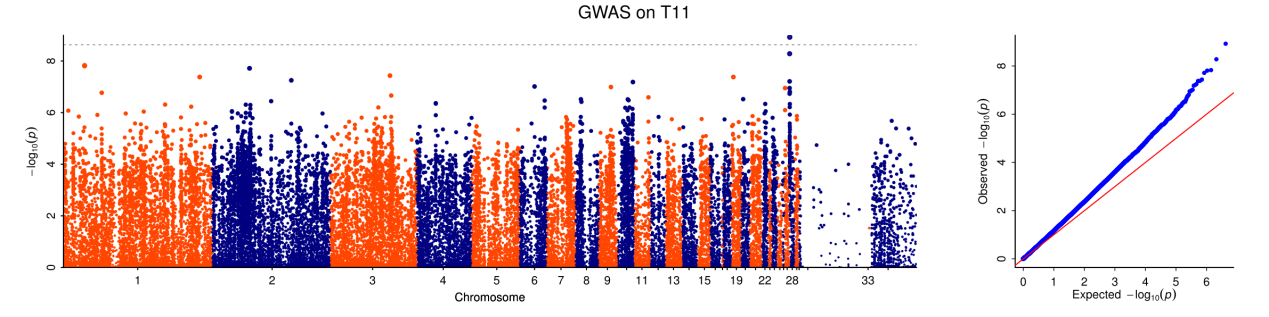


Figure S14 GWAS based on 100 days old.


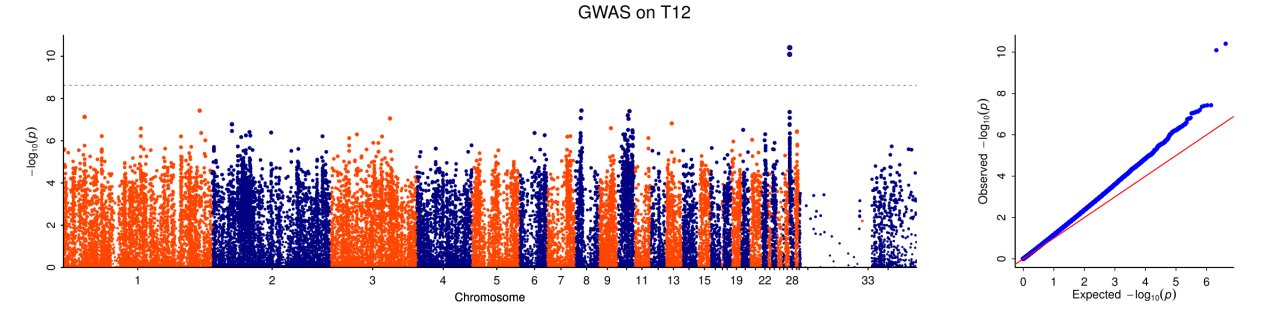


Figure S15 GWAS based on 110 days old.
